# Current Advances in Lung Ultrasound in COVID-19 Critically Ill Patients: A Narrative Review

**DOI:** 10.3390/jcm11175001

**Published:** 2022-08-26

**Authors:** Minh Pierre Lê, Mathieu Jozwiak, Driss Laghlam

**Affiliations:** 1 Service de Médecine Intensive-Réanimation, Hôpital Cochin, Assistance Publique-Hôpitaux de Paris (AP-HP), Centre & Université Paris Cité, 75014 Paris, France; 2 Service de Médecine Intensive Réanimation, Centre Hospitalier Universitaire de Nice, Hôpital l’Archet 1, 151 Route Saint Antoine de Ginestière, 06200 Nice, France; 3 UR2CA, Unité de Recherche Clinique Côte d’Azur, Université Côte d’Azur, 06200 Nice, France

**Keywords:** lung ultrasound, LUS score, COVID-19 pneumonia, critically ill patients

## Abstract

Lung ultrasound (LUS) has a relatively recent democratization due to the better availability and training of physicians, especially in intensive care units. LUS is a relatively cheap and easy-to-learn and -use bedside technique that evaluates pulmonary morphology when using simple algorithms. During the global COVID-19 pandemic, LUS was found to be an accurate tool to quickly diagnose, triage and monitor patients with COVID-19 pneumonia. This paper aims to provide a comprehensive review of LUS use during the COVID-19 pandemic. The first section of our work defines the technique, the practical approach and the semeiotic signs of LUS examination. The second section exposed the COVID-19 pattern in LUS examination and the difference between the differential diagnosis patterns and the well-correlation found with computer tomography scan findings. In the third section, we described the utility of LUS in the management of COVID-19 patients, allowing an early diagnosis and triage in the emergency department, as the monitoring of pneumonia course (pneumonia progression, alveolar recruitment, mechanical ventilation weaning) and detection of secondary complications (pneumothorax, superinfection). Moreover, we describe the usefulness of LUS as a marker of the prognosis of COVID-19 pneumonia in the fourth section. Finally, the 5th part is focused on describing the interest of the LUS, as a non-ionized technique, in the management of pregnant COVID-19 women.

## 1. Introduction

Lung ultrasound (LUS) was first described in the 80s but has a relatively recent democratization due to better availability and training of physicians, especially in intensive care units (ICUs). LUS is a relatively cheap and easy bedside technique for evaluating pulmonary morphology. Moreover, when using simple algorithms, it is an easy-to-learn and -use technique. Recently, in view of the global COVID-19 pandemic with massive influx of patients in the emergency department and in the ICU, physicians have been looking for accurate tools to quickly diagnose, triage and monitor patients with COVID-19 pneumonia. Considering its availability in many settings, including low-income environments and the restricted access to chest imaging by computer tomography (CT) scan, LUS has seen its use spread and democratized around the world. Indeed, the diagnostic accuracy of LUS in comparison with CT for the early management of COVID-19 patients is paramount. Subsequently, different fields of application of LUS in COVID-19 pneumonia were described, such as lung lesion progression monitoring, prognosis evaluation or detection of lung secondary complications.

**Figure 1:**
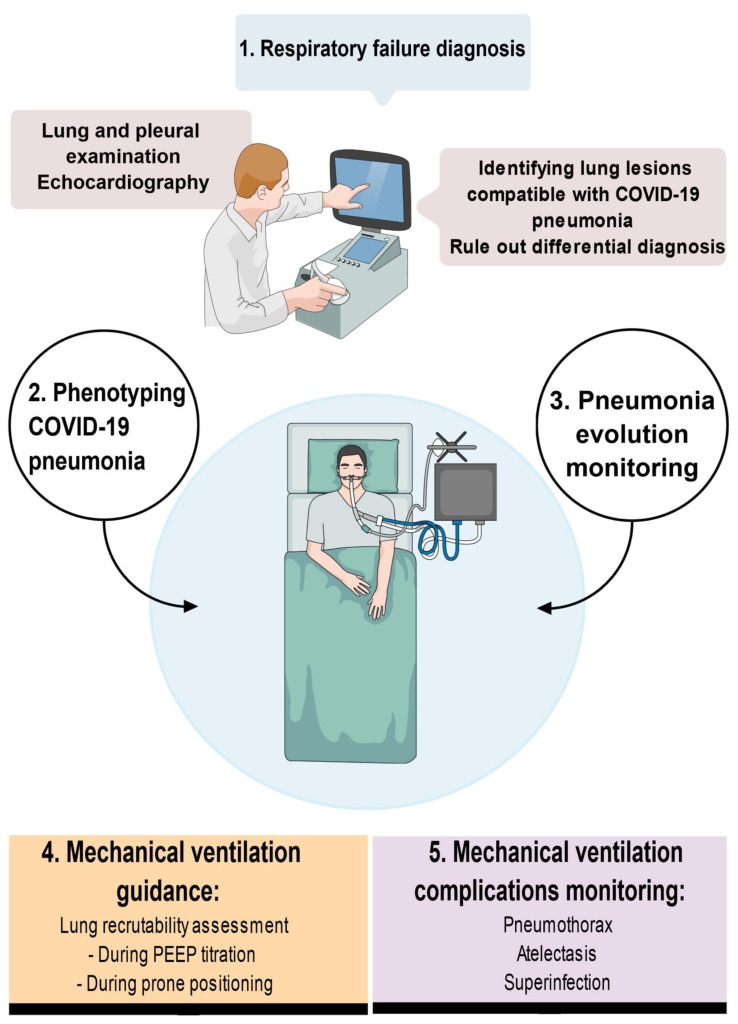
Scope of lung ultrasound in patients admitted with acute respiratory failure and suspected COVID-19 pneumonia.

In this review, we aimed to depict the current advances in LUS use in COVID-19 critically ill patients, describing the technique, ultrasound patterns and its value in the management of patients (see Figure 1).

## 2. Technique and Practical Approach

### 2.1. Which Probe to Choose and How to Use It?

**Figure 2:**
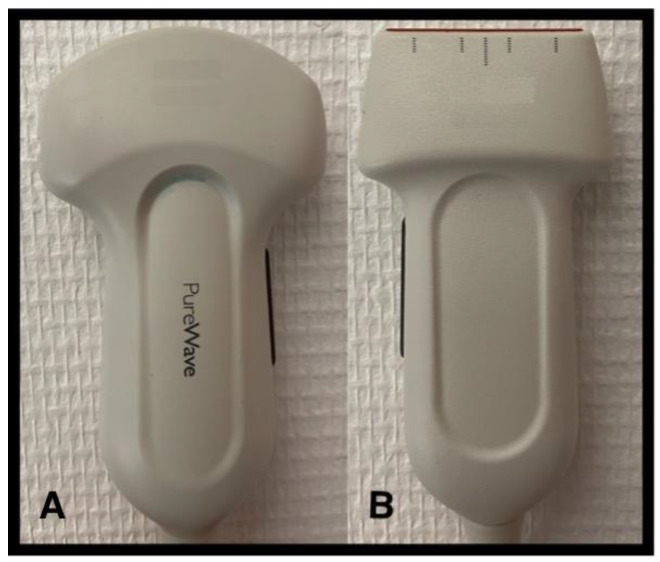
Probes used for lung ultrasound examination. (**A**). Convex probe, low frequency (3–5 Hz), preferred to explore deeper abnormalities. (**B**). Linear probe, high frequency (8–12 Hz), has a high superficial definition and low penetration capacity and allows better detection of superficial abnormalities with excellent images of the pleura and lung sliding.

All probes usually available in critical care settings can be used to perform LUS. The linear probe (see Figure 2), due to its high frequency (8–12 MHz), has a high superficial definition and low penetration capacity and allows better detection of superficial abnormalities with excellent images of the pleura and lung sliding (pneumothorax, parietal abnormality, sub pleural consolidation, etc.). The convex probe (see Figure 2), low frequency (3–5 MHz), allows better detection of deeper abnormalities (condensation, interstitial and alveolar syndrome, diaphragmatic movement) because of its good penetration and large sector width. However, because of the large footprint, the convex probe requires some angulation when analyzing the postero-lateral area to avoid the ribs. Phase array transducers (3–4.5 MHz) could be useful footprints for getting between the ribs. They can also be used to demonstrate all signs of LUS, but the clarity of the images is not as good.

In practice, linear and convex probes allow the detection of the same lung artefacts and lesions in M-mode (time-motion mode). The M-mode records displacement of mobile structures according to the time, in particular of pleura, also known as pleural shift. Graphic recording on a line of exploration is represented in ordinate, according to the time, which scrolls in abscissa. 

The probe can be positioned longitudinally, perpendicular to the ribs, and obliquely along the intercostal spaces. The longitudinal approach allows visualization of the upper and lower ribs with, a little deeper, the parietal pleura (the so-called “bat-sign”). The oblique approach allows visualization of a larger part of the pleural line, which is not interrupted by rib shadows.

### 2.2. Windows

#### 2.2.1. Windows

**Figure 3:**
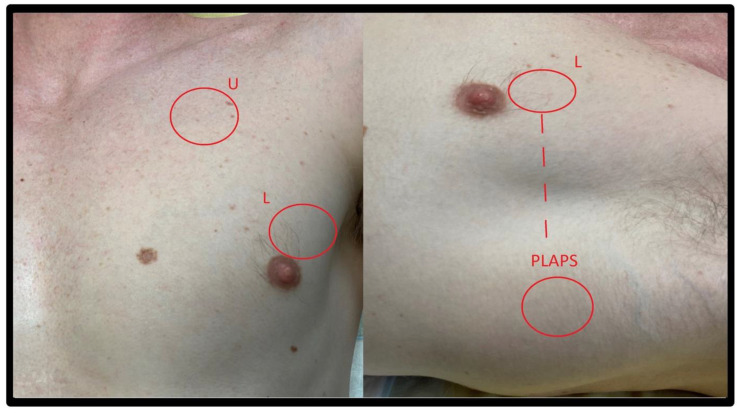
BLUE Protocol chest areas. Three areas of interest to explore according to BLUE protocol U: The upper BLUE-areas is at the middle of a virtual hand placed along the clavicula. L: The lower BLUE-areas is at the middle of the palm of another virtual hand placed below the first one. PLAPS: The PLAPS-areas (PosteroLateral Alveolar and Pleural Syndrome) is placed in the horizontal continuation of the lower point in the posterior axillary line.

**Figure 4:**
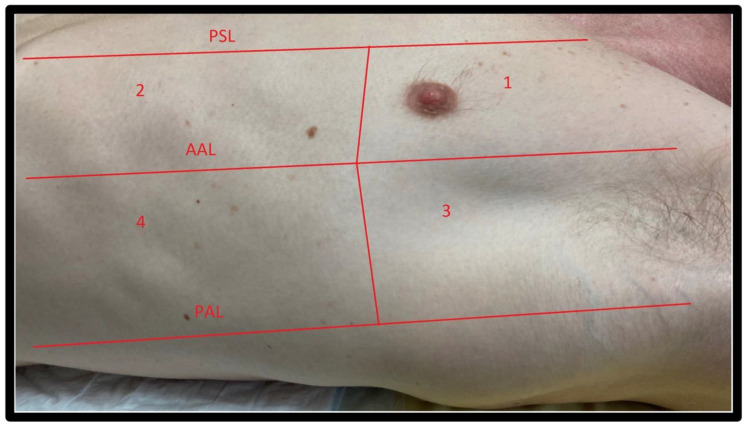
International Consensus Conference for point-of-care lung ultrasound areas. Four chest areas per side considered for complete eight-zone lung ultrasound examination (modified from Volpicelli et al. [5]). AAL: Anterior Axillary Line; PAL: Posterior Axillary Line; PSL: Parasternal line.

**Figure 5:**
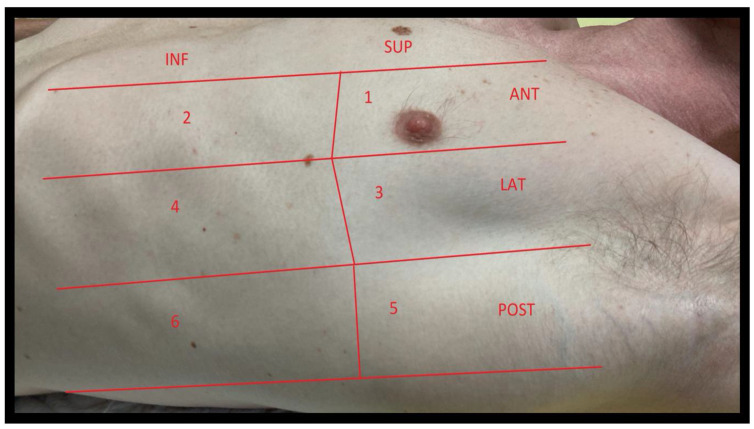
Six areas per hemithorax approach for LUS examination in ICU. Each hemithorax is divided into three areas, using anterior and posterior axillary lines (anterior, lateral and posterior). Each area is divided into two areas: superior and inferior. The LUS score is calculated on a total score of 36 (each area is evaluated as 0 to 3). ANT: Anterior; LAT: Lateral; POST: Posterior; SUP: Superior; INF: Inferior.

Several standardized protocols have been proposed for LUS examination in the emergency department and ICU. The most commonly used are the Bedside Lung Ultrasound in Emergency (BLUE) Protocol (three areas of interest per lung, (see Figure 3)) [1], a four areas per lung approach derived from the international consensus conference recommendations for point-of-care LUS established in 2012 (see Figure 4) [2]; and a more extensive six areas per lung protocol used preferentially in ICU, See Figure 5 [3]. In the COVID-19 setting, the CLUE (COVID-19 LUS in the emergency department) protocol has also been proposed [4].

**Figure 6:**
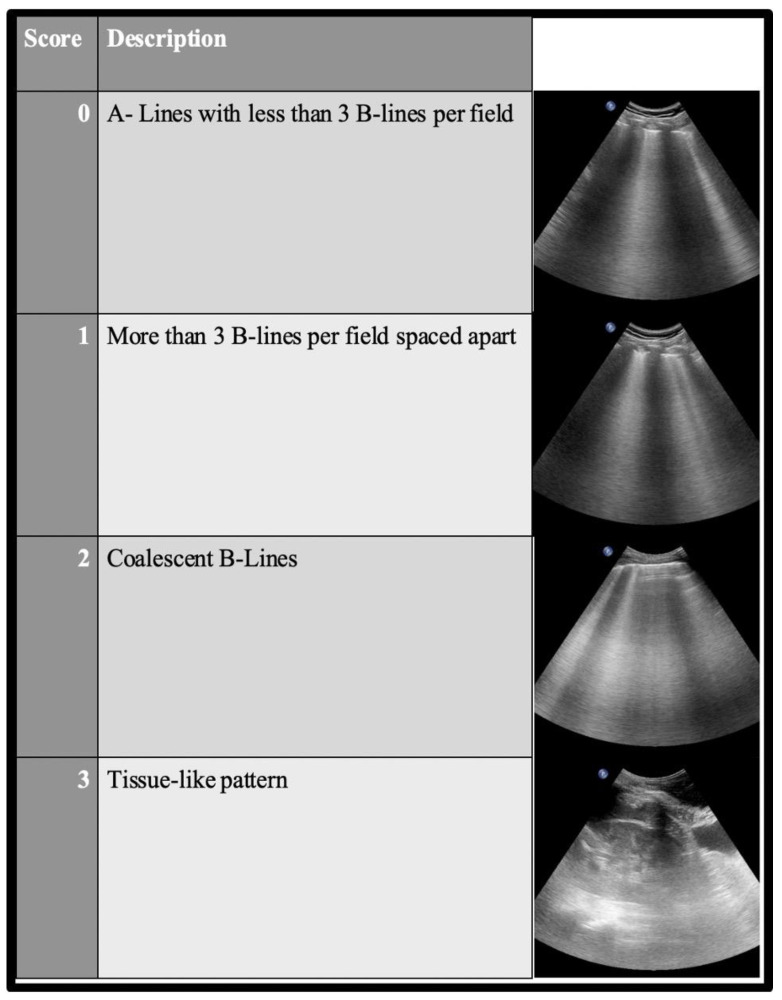
LUS score. Each chest area of interest must be evaluated from 0 to 3. The total score ranges from 18 (BLUE protocol, 3 areas of interest per lung) to 24 (ICC, 4 areas of interest per lung) or 36 (six areas per lung approach used preferentially in the ICU).

The LUS score is applied according to LUS findings, allowing an evaluation of the initial severity and a monitoring of the evolution of pneumonia. Each zone of interest must be evaluated from 0 to 3, with a total score ranging from 18 to 36 according to the use of a BLUE, ICC protocol or six areas per lung approach, respectively. (See Figure 6). Most of the studies cited in this review used an LUS score based on six areas per lung approach (total score of 36); it would be notified when another LUS score was applied.

#### 2.2.2. LUS Semeiotic Signs

Ultrasounds are not transmitted through a normally aerated lung, and only the pleural line can be seen. The air and bones interrupt the transmission of ultrasound beams. Consequently, LUS is limited to the study of pleura and subpleural lesions through intercostal spaces. Because of the difference in acoustic impedance between air in the lung and superficial tissues, ultrasound cannot penetrate the lung, and artifacts are generated by the pleura.

The most important thing to understand in the interpretation of LUS is that ultrasound findings are made up of both artifacts (normal and pathological) and real images (always pathological and visible only in the absence of air interposition).

##### Artifacts

**Figure 7:**
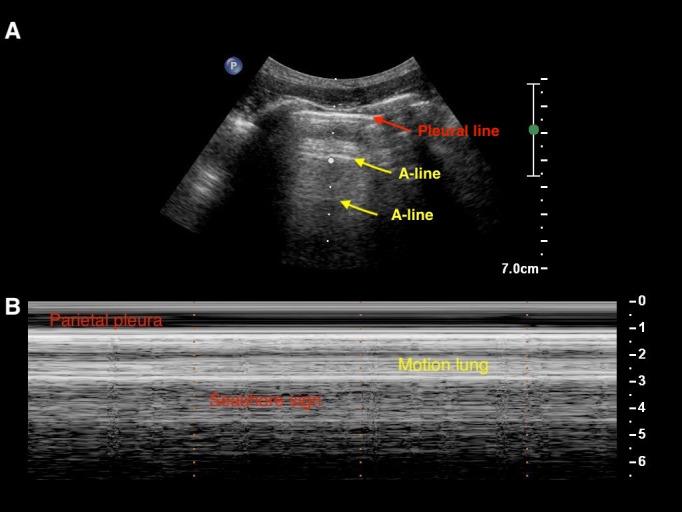
Basic signs of lung ultrasound in B- and M-mode, normal lung. (**A**) In B-mode, the red arrow indicates the pleural line (horizontal echogenic line under subcutaneous tissue). The pleural line can be observed moving with respiratory movement. The yellow arrows represents the horizontal A-lines. (**B**) The M-mode demonstrates normal pleura sliding: subcutaneous tissues above the pleural line do not move away or toward the probe and are represented as straight lines. The pattern below the pleura is an artifact deriving from visceral pleura sliding, as it generates a sandy pattern called the “seashore sign.”

The first aim of LUS is to detect pleural shift with the identification of the pleural line and lung sliding, which means visualization in B-mode (dynamic mode) of the horizontal movement of the parietal pleura below two successive ribs. The pleural line appears as a thin, echogenic line seen just deep into the intercostal musculature between the ribs. A normal pleural line measures <0.2–0.3 mm. The association of the visualization of the two ribs with the parietal pleura is called a bat sign. At the same location, the M-mode allows for a more precise analysis of lung sliding. In M-Mode, the “seashore sign” is derived from the difference of aspect of subcutaneous tissues above the pleural line that do not move away or toward the probe and are represented as straight lines and the pattern below the pleura, which is an artifact deriving from the visceral pleura sliding (See Figure 7).

**Figure 8:**
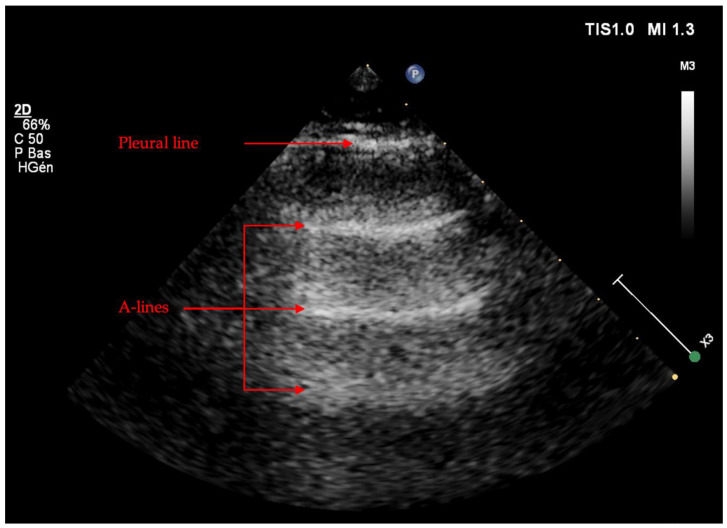
A-lines. A-lines represent horizontal parallel artefacts behind the pleural line at multiples of distance of the probe, indicating a good lung aeration (longitudinal view, linear probe).

A-lines are a normal basic artefact represented by a repeated horizontal long path form of reverberation, caused by reflections of the pleura, behind the pleural line at multiples of distance of the US probe (see Figure 8). It is an ultra-sonographic artifact indicating a normal lung aeration, which represents the visceral–parietal pleural interface. This indicates that gas is present, reflecting the ultrasound waves back to the probe. 

**Figure 9:**
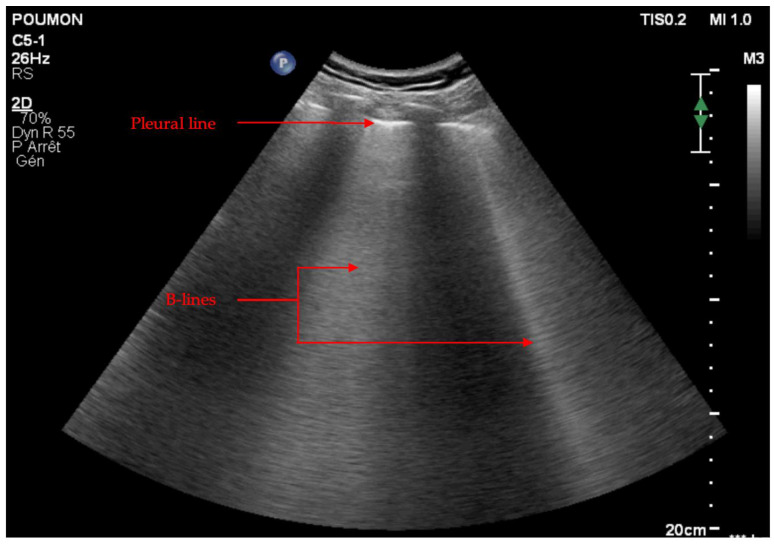
B-lines. B-lines appear as vertical hyperechoic line artefacts taking the whole height of the screen crossing the A-lines without decreasing in intensity. B-lines always arising from the pleural line and moving simultaneously with lung sliding and normal lungs can demonstrate up to three B-lines per lung window/intercostal space.

B-lines are another form of reverberation artifact in which bright “comet tail”-like lines are seen extending from the pleural line to the bottom of the screen, obliterating A-lines (See Figure 9). In other terms, B-lines correspond to vertical hyperechoic line artefacts taking the whole height of the screen crossing the above-mentioned A lines without decreasing in intensity. B-lines always arising from the pleural line and moving simultaneously with lung sliding and normal lungs can demonstrate up to three B-lines per lung window/intercostal space.

Multiple B-lines in one of the previously described application areas must be counted between 0 and 10. The presence of more than three B-lines in a chest area of interest, also called a “B-pattern,” indicates an interstitial syndrome. 

##### Real Images

Real images seen in the LUS are always pathological and visible only in the absence of air interposition. Lung consolidation is either translobar or not, and this characteristic is associated with specific ultrasound patterns [6]. Non-translobar consolidations are described as small subpleural, hypoechogenic images with deep irregular boundaries called “fractal “or “shred sign,” while translobar consolidations give a tissue-like pattern and an image shaped like an anatomical lung. The air bronchogram is visualized as hyperechogenic intraparenchymal images with a shape that can be punctiform or linear/arborescent. In addition, direct visualization of a pleural effusion is possible, with the identification of a hypo- or anechoic area over the echogenic curvilinear diaphragm. 

##### Pneumothorax

**Figure 10:**
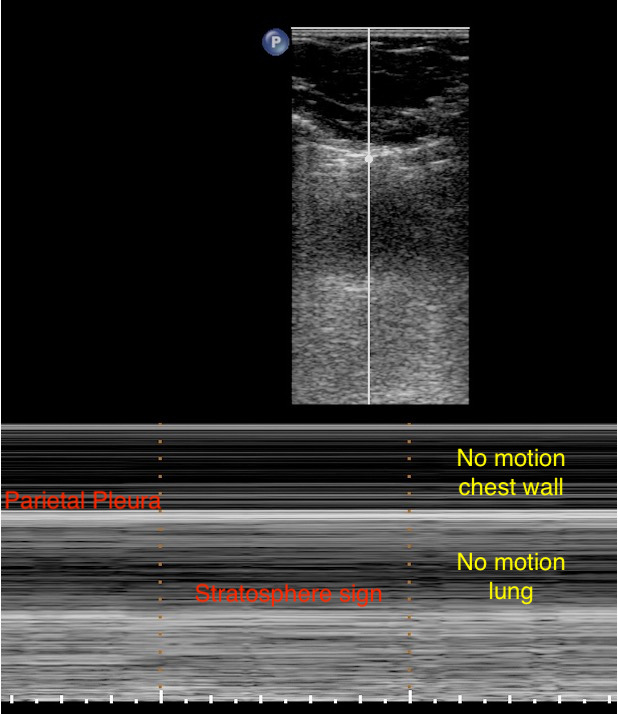
Pneumothorax in M-mode. Parietal pleura visualized below two successive ribs. There was an absence of pleural sliding and seashore signs in M-mode, which indicated the presence of gas effusion. In M-mode, the stratosphere sign and straight horizontal lines above and beneath the pleural line can also be observed, representing the absence of pleural sliding.

The absence of pleural shift and the visualization in TM mode of a stratosphere sign, also called “lung point,” visualized when the visceral pleura begins to separate from the parietal pleura, indicates with excellent sensitivity the existence of a gas effusion, e.g., pneumothorax (see Figure 10). In this case, the pleural sliding was only in an anteroposterior axis in interaction with the heartbeat. Lichtenstein et al. showed that the association of a lung point with no pleural shift and B-lines combined with lung pulse confirmed the diagnosis of pneumothorax with an excellent specificity of nearly 100% [7]. Thus, current guidelines emphasize strong recommendations for using LUS to diagnose pneumothorax, with more accuracy than chest X-ray [2]. 

##### Patterns in the LUS Examination

All of these signs could be associated with building up different profiles to be applied in patients presenting with acute respiratory failure. Consolidations visualized through an A-profile (A-lines with less than 3 B-lines per field) are associated with the presence of a lung focus [8]. The presence of many B-lines found bilaterally, symmetrically and homogeneously allows the diagnosis of acute pulmonary edema to be made as soon as the patient is taken to the emergency room [9]. The number of B-lines in this context allows acute pulmonary edema to be quantified and graded [10]. The presence of B-lines or consolidation unilaterally could orientate the diagnosis toward bacterial pneumonia. These consolidations will appear transpolar or sub pleural with inhomogeneous A lines, decreased pleural sliding and irregular pleura.

### 2.3. Learning Curve and Inter- and Intra-Individual Agreement

LUS can be used by different physicians (emergency, intensivist, etc.) and is an easy-to-learn technique. Chiem et al. showed in a single-center prospective study on 380 patients that emergency medicine residents (*n* = 66), who received a 30-min training course, can detect B-lines with a sensitivity of 85% and specificity of 84% when compared to an emergency LUS expert. This study showed that only a few practices are needed for novice physicians to detect basic sonographic signs [11]. Additionally, LUS was found to be reproducible and reliable, showing excellent intra- and inter-agreement in a cohort of 91 patients; evaluated by two consecutive LUS by two experts (>100 LUS performed) or one expert and one novice (>5 LUS performed). Both pairs (Expert/expert or expert/ novice) showed a good agreement, in particular to the anterior/superior thoracic zones (ICC: 0.904 and 0.777 to expert/expert, ICC: 0.862 and 0.834 to expert/novice). Reinterpretation of the randomized video clip also showed good agreement of 0.697 and 0.647, respectively, with the 2 experts [12]. 

These studies showed that novice physicians could, with few practices, easily detect basic sonographic signs (such as B-lines or a large condensation) or calculate the LUS score. However, it seems not easy to learn how to obtain meaningful images or to interpret the LUS findings when they appear combined. LUS was not always considered an easy examination, as it requires expertise in pattern recognition and has a steep learning curve. The practitioner needs to be able to interpret normal and abnormal appearances to detect lung disease. Before the COVID-19 pandemic, in critically ill patients, Rouby et al. suggested that 25 ultrasound examinations, supervised by a physician with expertise in bedside LUS, could be a reasonable requirement to measure the LUS score, including appropriate recognition of normal aeration, interstitial syndrome, alveolar edema, and lung consolidations [13]. 

Classical LUS education using didactic teaching and hands-on training was challenging by a rapid expansion in the number of patients admitted for COVID-19 pneumonia, leading to a high number of practitioners who needed to master the technique in a short period of time [14,15]. Therefore, during the COVID-19 period and beyond, there is still some concern about how structured learning of this technique must be. 

Potential learning techniques should be combined and included: didactic lectures, in vitro and in situ (on healthy volunteers and then on patients) simulation [14].

### 2.4. Usefulness as a Non-Irradiated Imagery Technique

Lung ultrasound could assist in the diagnosis of patients with acute respiratory distress in emergency departments. LUS takes advantage of CT scans or chest radiography in intensive care units due to less radiation. A single retrospective monocentric study in Italy showed that since LUS was introduced daily in the ICU, there was a drastic reduction of average chest radiography among 4134 patients (0.97 per patient vs. 0.42 with daily LUS), with an estimated cost reduction of 57%, without affecting patient outcome [16]. The daily use of LUS must be discussed compared to the frequent use of ionizing radiation, as the long-term effects of these techniques could lead to neoplasia due to stochastic effects and deterministic effects.

## 3. Diagnosis of COVID-19 Pneumonia

The fast progression of COVID-19 has required early screening, detection and timely monitoring by imaging modalities. COVID-19-related pulmonary lesions preferentially involved the subpleural regions with frequent diffuse damage, making them easily accessible to ultrasound exploration. 

### 3.1. COVID-19 Pattern in LUS Examination

LUS findings during COVID-19 pneumonia have been rapidly described [17].

**Figure 11:**
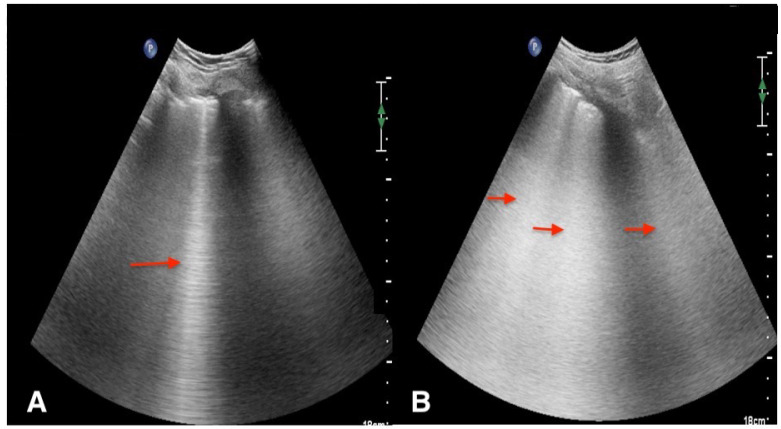
Interstitial syndrome with B-pattern in a patient with COVID-19 pneumonia. (**A**). One isolated B-line (red arrow) visualized on the upper areas bilaterally (longitudinal view, convex probe). (**B**). In the same patient, multiple coalescent B-lines are visualized in the lower area bilaterally (red arrows) arising from the pleural line and spreading up to the edge of the screen, representing interstitial involvement “B-pattern” (longitudinal view, convex probe). This corresponds to severe impairment in lung aeration resulting from partial filling of alveolar spaces by pulmonary edema.

**Figure 12:**
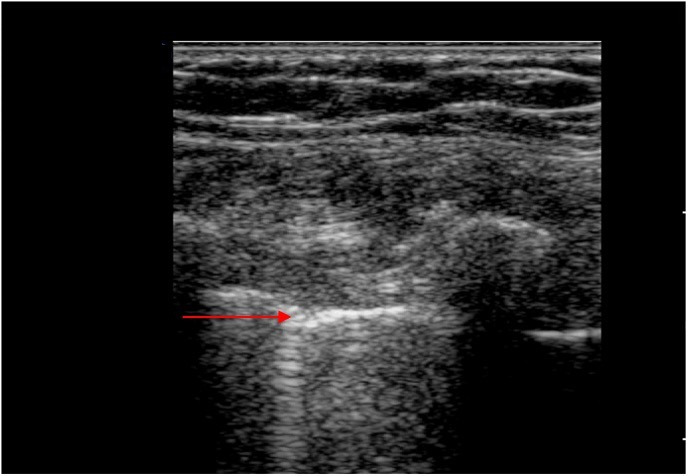
Thickened pleural lines in a COVID-19 patient. Thickened and irregular pleura (red arrow), suggestive of interstitial lung disease.

**Figure 13:**
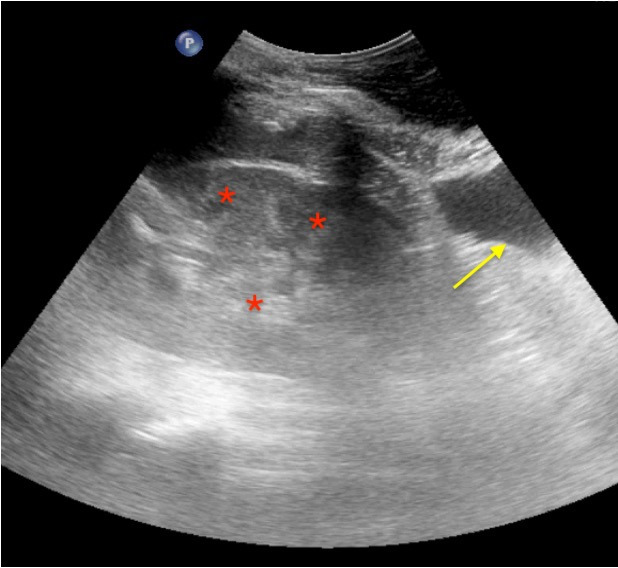
Lung consolidation in a COVID-19 patient. Lobar consolidations (translobar) visualized as a tissue-like pattern of the lower lobe. The air bronchograms are visualized as hyperechoic signs within consolidation (air-filled bronchi) (red Asterix). A small pleural effusion is associated (yellow arrow).

Overall, in patients with COVID-19 pneumonia, LUS revealed a typical pattern of diffuse interstitial lung syndrome, characterized by multiple or confluent bilateral B lines with spared areas (see Figure 11), thickening of the pleural line with pleural line irregularity (see Figure 12) and peripheral consolidations (see Figure 13). However, specific signs of COVID-19 in LUS were rare. 

In detail, LUS signs associated with COVID-19 pneumonia were similar to viral pneumonia’s signs, with indirect signs pointing toward B-lines, irregular pleural lines, and pleural effusion [18]. Nevertheless, none of these signs were specific or pathognomonic to COVID-19 pneumonia. COVID-19 pneumonia lesions were characterized by initial interstitial involvement, with a bilateral posterior peripheral and preferential sub pleural distribution. The most common artefact was interstitial involvement with B-patterns. In the emergency department, when LUS was used in patient triage in combination with medical history and emergency physician estimate of high clinical probability, the presence of bilateral B-lines on LUS was associated with a higher positive likelihood ratio of final COVID-19 diagnosis defined by positive SARS-CoV-2 RT-PCR (7.09 (95%CI 2.77 to 18.12). On the other hand, patients with a low clinical probability of COVID-19 with absence of B-lines had a low negative likelihood ratio of positive SARS-CoV-2 RT-PCR (0.26, 95% CI 0.15 to 0.45) [19]. 

Alveolar syndrome could also be visualized by B-line clusters with consolidations and air bronchograms representing probable bacterial surinfection. This involvement could be detected by the increasing number of B-lines over time, corresponding to pneumonia extension. These B-lines could become coalescent and go as far as consolidation or even hepatization of the lung, corresponding to a lung with little or no ventilation. As described in the CT scan, irregularity of the pleura or sub pleural consolidations might be observed in LUS, with thickened pleural lines [17]. The use of a high-frequency probe seemed to be more accurate for visualization of pleural irregularities, whereas a low-frequency probe seems to be more suitable for visualizing sub pleural consolidations. 

A new sign artifact, named a “light beam,” was often described on COVID-19 pneumonia, corresponding to the early appearance of “ground glass,” which might also be visualized in CT scan [18]. It was described as a vertical, band-shaped artifact, appearing and disappearing rapidly from the screen with an « on-off » effect. This artefact arises from a regular pleural line between the separated B-lines and normal areas. A single-center study showed that multiple light beams were found in 48 patients among 49 COVID-19 positive patients. However, data concerning the sensibility and specificity of this sign remain scarce.

After identifying these LUS signs in the early hours of the pandemic, Volpicelli et al. proposed separating patients into four different patterns according to the probability of COVID-19 disease (A-low probability, B-Pathological finding on LUS, C-Intermediate probability, D-High probability) to assess or help diagnosis of COVID-19 pneumonia [18].

Finally, although the sensitivity of LUS is relatively high, its specificity may not be the same. In the post-epidemic era, it could be difficult to distinguish COVID-19 from other causes of pneumonia. Below, we describe the potential differential diagnosis in patients with acute respiratory distress and their pattern in LUS examination.

### 3.2. Differential Diagnosis from Other LUS Patterns

LUS is often used in the differential diagnosis of acute respiratory failure. The differential diagnosis of patients suspected of having COVID-19 pneumonia could be challenging in the post-COVID-19 period because other acute conditions could be involved, such as cardiogenic and non-cardiogenic pulmonary edema or bacterial pneumonia.

LUS must be integrated in a diagnosis algorithm with the patient’s medical history, physical examination, the time lapse between the examination and the day of symptom onset and other findings.

However, each condition presents some peculiarities that, as described below, could at least orientate the diagnosis [20,21]. Cardiogenic pulmonary edema LUS findings usually show multiple diffuse, bilateral B-lines, which are separated in the earlier phases and tend to be more confluent and numerous in more advanced cases. Conversely to other causes, in cardiogenic pulmonary edema, B-lines tend to follow a homogenous, gravity-related distribution over the chest. Furthermore, the pleural appears thin and regular, while small consolidations are rare.

Diagnosis of bacterial pneumonia in LUS might involve consolidations of different sizes, quite large and with a tissue-like appearance. This aspect was not typically present in the early phase of COVID-19 pneumonia. Additionally, LUS could also show B-lines in one area of the chest, which represented a focal interstitial syndrome multiple, indicating either consolidation not yet established in the lung parenchyma or perilesional edema or consolidation that is not peripheral enough to be seen by LUS. Typically, an isolated large lobar consolidation with dynamic air bronchograms orients to bacterial pneumonia. Moreover, as COVID-19 pneumonia does not seem to be associated with complex pleural disease, a large pleural effusion with atelectatic consolidations would suggest another infection.

Chronic diffuse interstitial pulmonary are more likely associated in LUS examination with diffuse abnormalities of the pleural line (irregularity and fragmented aspect of pleural line) and multiple bilateral B-lines with no patchy distribution. In these pathologies, peripheral consolidations and pleural effusions are rare.

Finally, the most difficult differential diagnosis is non-cardiogenic pulmonary edema, e.g., aRDS from causes other than COVID-19. The LUS pattern of patients with non-COVID-19-related ARDS associated patchy and non-gravity-related distribution B-lines, irregularity of pleural lines, and consolidations of different sizes, from small peripheral consolidations to larger ones with a tissue-like appearance. The relatively good respiratory tolerance discordant with extensive lesions on LUS could, in this situation, point to a diagnosis of COVID-19 pneumonia.

### 3.3. Correlation between Lung Ultrasound and CT

Chest CT scans have been predominantly used for COVID-19 diagnosis. However, limitations, including radiation exposure, the safety of transferring patients for CT scans, contamination of the medical devices and nosocomial spreading of the virus, limited mobility and resource consumption, could restrict its use. Since the beginning of the pandemic, a good agreement between CT and LUS findings has been found. Given its portability and safety, LUS is a possible alternative to ionizing radiation imaging, especially in resource-limited environments.

First, the LUS score was found to be significantly associated with thoracic CT scan findings and hypoxemia severity. In a prospective study of 100 patients, including patients who had an LUS exam at admission to the emergency department or ICU (within the first 2 h after admission), as well as a chest CT scan within the 24 h following admission, Zieleskiewicz et al. reported that the LUS score was significantly associated with pneumonia severity assessed by chest CT and clinical features. The AUC of the ROC curve of the relationship between LUS and chest CT scan for the assessment of severe SARS-CoV-2 pneumonia was 0.78 (CI 95% 0.68–0.87; *p* < 0.0001) [22]. Moreover, an LUS score > 23 predicted severe SARS-CoV-2 pneumonia diagnosed by chest CT scan with a specificity > 90% and a positive predictive value of 70% in 23 patients (23%), while an LUS score < 13 excluded severe SARS-CoV-2 pneumonia diagnosed by chest CT scan with a sensitivity > 90% and a negative predictive value of 92% in 39 patients (39%). However, thirty-eight patients (38%) were in the gray zone. The strength of this study was the close timing between admission, LUS and CT scan examinations, giving a great description and correlation of COVID-19 severe lesions assessed with both techniques [22].

Another study performed in China on 29 patients with 45 simultaneous LUS + CT imaging data (defined as exam interval ≤ 12 h) showed a better sensitivity of LUS compared to CT scans in the detection of COVID-19 lesions. Yang et al. reported that LUS was more sensitive than chest CT in the diagnosis of regional alveolar-interstitial pattern (AIP, defined as multiple B-lines (≥3) shown within a region by LUS and as the presence of ground glass opacity pattern by CT; 60% vs. 38.5%, *p* < 0.0001); alveolar-interstitial syndrome (defined as positive AIP regions (≥2) per side and bilateral positivity; 93.3% vs. 68.9%, *p* = 0.001), consolidation (38.9% vs. 3%, *p* < 0.0001) and pleural effusion (74.4% vs. 15.6%, *p* < 0.0001) [23]. Finally, De Alencar and colleagues confirmed in a prospective study including 180 patients that the LUS score in the emergency department was correlated with findings from thoracic CT scans, and they also found that the LUS score could reliably predict the estimated extent of parenchymal involvement [24].

Overall, these findings suggest that LUS could be a reliable alternative to thoracic CT scans to assess the severity of pneumonia in patients with COVID-19 pneumonia.

Despite the good correlation described above between CT scans and LUS in patients with COVID-19 pneumonia, one of the main concerns was the difficulty of differentiating the cause of interstitial syndrome by LUS. Indeed, in a patient presenting with acute respiratory failure, the interstitial syndrome identified by LUS could be related to another cause in a patient with other comorbidities (acute cardiogenic pulmonary edema, chronic interstitial lung disease, sequelae of infections, etc.). In such a situation, it could be difficult to attribute acute respiratory distress to COVID-19 pneumonia or decompensated previous illness.

## 4. LUS Use in the Management of COVID-19 Patients

### 4.1. Early Management of COVID 19 Patients: Identification and Triage

The continued influx of patients suffering from COVID-19 severe acute respiratory syndrome in a resource-limited environment required rapid and efficient triage, which could be facilitated using LUS for the diagnosis and management of those patients. 

Combining LUS patterns with clinical phenotypes at presentation in the emergency department or in the ICU could rapidly identify patients with or without COVID-19 pneumonia, as demonstrated in an international multicenter observational study in 20 US and European hospitals [25]. Interestingly, a high LUS score on admission to the emergency department was associated with the need for ICU admission [24,26,27,28]. Thus, LUS could assist physicians in the emergency department in performing rapid diagnosis and prognostic stratification to make a quick decision on the patient’s destination, thus potentially shortening the delay to ICU admission [29,30,31,32].

In addition, a high LUS score on admission to the emergency department or to the ICU could reliably identify patients who were likely to fail non-invasive ventilation [27] and was associated with the need for ICU admission and invasive mechanical ventilation [22,24,27,33,34,35,36]. Thus, LUS might also help physicians in the decision-making process, allowing for earlier management and appropriate therapies [18,37,38]. However, it is important to keep in mind that although LUS may be an interesting tool to contribute to improving the prognosis of patients with COVID-19 pneumonia, LUS may not be conclusive for some of them [22].

### 4.2. Management of Critically Ill COVID-19 Patients

The contribution of LUS in the management of the COVID-19 pneumonia course could be multi-fold.

LUS can help physicians make a timely diagnosis of secondary pulmonary complications, guide and monitor alveolar recruitment in mechanically ventilated patients, and predict ventilator weaning failure. 

#### 4.2.1. Detection of Secondary Complications

In addition to the initial diagnosis and detection of disease progression, LUS could be useful for identifying the cause of secondary respiratory deterioration, especially in ventilated COVID-19-related ARDS. By adding LUS in the diagnosis toolset of mechanical ventilation complications in COVID-19-related ARDS, physicians could rapidly rule out differential diagnoses, such as pleural effusion, alveolar consolidation related to atelectasis or ventilator-associated pneumonia, and pneumothorax. First, loss of pleural sliding in reel mode with barcode/stratosphere sign in M-mode or identifying a lung point can indicate pneumothorax. Abolished lung sliding is found anteriorly in quite all significant cases in supine patients, with 95% sensitivity and 100% negative predictive value [39]. When time lung sliding is present, pneumothorax could therefore be confidently discounted [40,41]. However, it should be kept in mind that loss of pleural sliding can also be caused by atelectasis, apnea, inflammatory adherences or hyperinflation. Second, LUS could be accurate for identifying atelectasy. For example, Lock et al. described a sudden drop in oxygenation in an intubated COVID-19 patient with absent breath sounds over the left upper chest but with persistent left pleural sliding in LUS examination, excluding pneumothorax and finally caused by partial ateclectasy of the lung [42].

Furthermore, lung ultrasound could be accurate for monitoring disease progression. At the patient’s bedside, the evolution of the LUS score could be used daily to follow the evolution of COVID-19 pneumonia in these patients. Indeed, LUS variations seem correlated with disease severity. In a prospective study on thirty-three patients, with a median of 9 (6–14) LUS evaluations per patient (delay between LUS evaluations of 2.1 (1.7–4.2) days), the LUS score increased significantly over time in non-survivors compared to survivors and was significantly and negatively correlated to PaO2/FIO2 ratio (*n* = 278, r = −0.37, *p* < 0.0001). In addition, the authors suggested that LUS could detect ventilator-associated pneumonia, as they reported that the LUS score increased in 83% of ventilatory-associated pneumonia episodes [43].

#### 4.2.2. Monitoring Alveolar Recruitment

Another field of application of LUS in critically ill patients is the guidance and monitoring of alveolar recruitment.

In mechanically ventilated patients, the LUS score (following BLUE protocol) could be an accurate tool to assess regional and global lung aeration [44] but bedside LUS demonstrates conflicting results on accuracy to estimate PEEP-induced lung recruitment in a non-COVID-19 setting. Some authors found a good correlation between PEEP-induced lung recruitment as measured by pressure-volume curves and ultrasound reaeration score (Rho = 0.88; *p* < 0.0001) and between LUS reaeration score and PEEP-induced increase in PaO₂: Rho = 0.63; *p* < 0.05), while others did not report an association between LUS score variations and lung recruitment (R = 0.01; *p* = 0.67) [44]. Overall, as LUS cannot assess PEEP-induced lung hyperinflation, it must be combined with other methods for PEEP titration [45]. 

In a single-center French study that included 24 patients with COVID-19-related ARDS, the global LUS score was not significantly associated with alveolar recruitment (PEEP-induced lung recruitment was assessed by the Recruitment/Inflation (R/I) ratio with high and low PEEP set at 15 cm H_2_O (for 30 min) and 5 cm H_2_O, respectively). In contrast, in the high recruiter sub-population (R/I > 0.7) there was a significant decrease in the LUS score, especially in the lateral and posterior lung regions. These results were combined with the R/I ratio and pulmonary compliance, so a change in LUS alone can’t define recruitability [46]. Further studies are needed to evaluate the performance of LUS to measure alveolar recruitment.

Additionally, it has been suggested in some case reports that serial LUS could be useful in monitoring the progression and improvement of lung aeration under veno-venous ECMO in non- and COVID-19-related ARDS patients [47,48]. In the same way, in a Danish single-center study of 10 severe COVID-19 related ARDS patients requiring venous-venous ECMO, an increase in the LUS score (following of 4 areas per lung analysis) in these patients was significantly associated with decreased compliance, while a decreasing LUS score over the ARDS course was associated with a higher probability of weaning from ECMO [49]. 

#### 4.2.3. Mechanical Ventilation Weaning

Finally, considering the prolonged duration of mechanical ventilation in COVID-19 patients, LUS could be used in association with echocardiography before and during the ventilator weaning course. LUS has been reported to be helpful for diagnosing weaning-induced pulmonary oedema [50,51]. In non-COVID-19 patients, an increase in the number of B-lines ≥ 6 in four anterior regions during the spontaneous breathing test (versus baseline before the test) was predictive of WIPO with a sensitivity of 88% (64–98), a specificity of 88% (62–98) and an AUC of 0.91 (0.75–0.98) [50].

## 5. Prediction of the Prognosis of the COVID-19 Pneumonia Course

In addition to early diagnosis and management, LUS usefulness could be extended to predict adverse events during hospitalization and/or outcomes in patients with COVID-19 pneumonia. A high LUS score on admission to the emergency department or to ICU was associated with a higher incidence of respiratory failure and ARDS [34], higher levels of inflammatory, cardiac injury or coagulopathy biomarkers [28,34], a higher mortality rate [24,26,33,34,36] and a higher length of stay in ICU or hospital [28,35]. Of note, the LUS score was a better predictor of adverse events during hospitalization than clinical parameters such as age, comorbidities and lymphocyte count. Indeed, a LUS score >12 reliably predicted adverse events with a sensitivity of 90% and a specificity of 91% [34]. Thus, a high LUS score on admission could be associated with up to a 6-fold increase in the odds of poor outcomes in patients with severe acute respiratory syndrome COVID-19 pneumonia [52]. However, performing LUS only on admission may not be sensitive enough to reliably predict outcomes, as it has been shown that LUS examination at 72 h but not on admission is associated with patient outcomes [53]. Therefore, serial LUS examinations during the ICU or hospital stay, rather than a single LUS examination on admission, could be an interesting prognostic tool. Lichter et al. prospectively showed in 120 patients hospitalized for COVID-19 pneumonia that increases in LUS scores during follow-up were associated with clinical deterioration in patients, regardless of the cause of the deterioration [33]. Similarly, other studies found that the evolution of the LUS score during the ICU stay in patients with COVID-19-related ARDS was closely related to the evolution of the ARDS severity and to patient outcomes, with a decrease in survivors and an increase in non-survivors of the LUS score during the ICU stay [43,54,55]. Similar findings were found in patients with COVID-19-related severe ARDS requiring venovenous extracorporeal membrane oxygenation [49] and in patients with non-severe or mild disease [56,57,58]. Finally, in a prospective multicenter study including 469 patients with COVID-19, Torres-Macho et al. assessed the prognostic accuracy of a serial LUS protocol [38]. The primary composite endpoint included death or the need for invasive mechanical ventilation. They found that the LUS score on admission to hospital combined with no increase in the LUS score on the second examination 72 h later ruled out death or the need for invasive mechanical ventilation. Conversely, an LUS score on admission to hospital combined with a significant increase in the LUS score on the second examination 72 h later reliably detected patients at high risk of death or need for invasive mechanical ventilation [38]. Thus, LUS appears to be useful for repetitive assessments and for monitoring disease evolution in patients with COVID-19 pneumonia, regardless of the severity of the disease [29,30].

Consistently, it has been shown that there was a closed relationship between histopathological analysis and post-mortem LUS findings in patients deceased from severe COVID-19-related ARDS, in whom LUS and minimally invasive autopsies were performed simultaneously [59]. In 28 patients, a significant correlation was found between pulmonary consolidation and diffuse alveolar damage, which is the histopathological hallmark of ARDS, and the LUS score associated with other variables could predict the proportion of pulmonary parenchyma with diffuse alveolar damage with reasonable reliability [59].

## 6. LUS Use in the Management of Pregnant COVID-19 Women

The overall risk of COVID-19 pneumonia in pregnant women is low. However, women who are pregnant or were recently pregnant are at increased risk of severe COVID-19 pneumonia. LUS may be of the most importance in this particular context, as ultrasound is a non-ionized technique; obstetricians and gynecologists are usually familiar with this technique [60,61,62]. Vetrugno and colleagues retrospectively assessed in 44 pregnant women the potential LUS differences between symptomatic and non-symptomatic women, the potential relationship between inflammatory biomarkers or oxygenation variables and the LUS score, and the potential relationship between the LUS score and clinical evolution during the ICU stay [63]. By repeating LUS on ICU admission 48 h and five days after admission, they found that symptomatic women had higher LUS scores than non-symptomatic women and that there was a significant correlation between LUS score and arterial oxygenation saturation as well as LUS score and C-reactive protein, interleukin-6 and pro-adrenomedullin. In addition, the evolution of the LUS score during the ICU stay was closely related to the evolution of the patients [63].

Similarly, LUS is used as a non-radiating technique in children and newborns. In this review, we have dealt only with its usefulness in adults, which is a missing part of the field of use of this technique and is a strength of this paper.

## 7. Conclusions

Lung ultrasound is an easy-to-use technique with a rapid learning curve that has been widely used during the COVID-19 pandemic. The role of LUS in adults with COVID-19 was consolidated during the last two years of pandemic, as its accuracy in COVID-19 pneumonia diagnosis permitted the inclusion of LUS into a standardized protocol, which helped in the early diagnosis and triage of COVID-19 patients. Furthermore, LUS could be a useful tool to monitor pneumonia progression and prognose illness severity. However, considering the low specificity of LUS, it could be difficult to distinguish COVID-19 from other causes of viral pneumonia in the post-epidemic era. The optimal use of LUS may be in the context of an extensive point-of-care ultrasound approach for patients with COVID-19, including heart, diaphragmatic and major vessel examination. 

## 8. Key Points

1. Lung ultrasound is a widely available, easy-to-learn bedside technique with a rapid learning curve.
2. Lung ultrasound allows us to rule in or rule out COVID-19 pneumonia combined with a compatible medical history by detecting alveolar or interstitial syndrome following the LUS score or the BLUE protocol.
3. Specific signs of COVID-19 are rare. Further studies are needed to assess the sensitivity and specificity of the « light beam » sign.
4. Lung ultrasound is helpful in screening, triage and treatment allocation in both the emergency and ICU departments.
5. The LUS score at admission could predict evolution toward pneumonia progression or death with moderate accuracy.
6. The LUS score could be used to monitor lung lesion progression and to detect complications in mechanically ventilated patients (pneumothorax and pleural effusion).
7. Little evidence exists regarding the utility of LUS in assessing alveolar recruitment efficiency.

## Data Availability

Not applicable.
